# How major international development organisations operationalise primary health care: a thematic content analysis of strategy documents

**DOI:** 10.1016/j.lanprc.2025.100014

**Published:** 2025-07

**Authors:** Luke N Allen, Erica Barbazza, Tova Tampe, Suraya Dalil, Shamsuzzoha Syed, Faraz Khalid

**Affiliations:** a Global Primary Care & Future Health Systems, Nuffield Department of Primary Care Health Sciences, University of Oxford, Oxford, UK; b WHO Special Programme on Primary Health Care, WHO, Geneva, Switzerland

## Abstract

Despite consensus around the need to prioritise primary health care (PHC), misaligned interpretations of this concept have real-world consequences for implementation. Here, we analysed how 30 major international development organisations operationalise PHC in their corporate strategy documents through thematic content analysis. The findings reveal that despite high-level endorsement for PHC, fewer than half of the reviewed documents explicitly mentioned PHC. Among those that did, PHC was conceptualised in varying ways: as a service delivery platform, level of care, bundle of interventions, or whole-of-society approach to health. From these conceptualisations, three different interpretations of PHC emerged—namely, the intended whole-of-society approach to health, strong or high-quality primary care, and selective or basic primary care. Integral components to PHC, including empowered people and communities and multisectoral action, were largely absent. These findings highlight the opportunities and urgency for improved alignment across international organisations to support a consistent approach aligned with the original vision of PHC.

## Introduction

The 1978 Declaration of Alma-Ata on primary health care (PHC) set out a broad vision for a comprehensive PHC approach to achieve Health for All.^1^ Its guiding principles included human rights; social justice; equity and solidarity; evidence-informed, context-responsive, and community-based care; participation, including self-reliance and self-determination; and intersectoral action. Recent years have seen an increasing awareness of the central importance of the PHC approach to health and wellbeing. The PHC approach serves as a catalyst in developing climate-resilient health systems, achieving universal health coverage, enhancing global health security, and strengthening pandemic preparedness.^2^

Global health and development organisations have increasingly embraced the PHC approach, as evidenced by multiple initiatives since the 2018 Astana Declaration^3^ and high-level policies such as the UN political declaration (RES/78/4)^4^ and Lusaka Agenda.^5^ Influential global health partners—including WHO, the World Bank, UNICEF, the Global Fund, Gavi, the Vaccine Alliance, and the Gates Foundation, together with multiple national and international aid agencies—now cite PHC as a strategic priority.

Despite this momentum, the implementation of PHC remains fragmented and inconsistent with its founding vision. As the WHO Director-General has observed, PHC remains one of the most important yet most misunderstood concepts in public health.^6^ Common characterisations of PHC encompass multiple dimensions, including a basket of interventions, a service delivery platform, and a philosophical approach to health.^7^ These overlapping and misaligned interpretations of PHC can make it difficult for health ministries, clinicians, policy makers, and communities to orient their roles, responsibilities, and actions. Although variation in implementation is to be expected considering differing organisational priorities and country contexts, a persistent pattern of misunderstanding, often shaped by narrow or reductionist interpretations, continues to undermine the original vision of PHC.

As we approach the midpoint of the 2030 Sustainable Development Goals timeline, 2025 presents an important opportunity to accelerate alignment across international organisations and national development agencies to reclaim the ambition that originally defined PHC. Current geopolitical instability places additional pressure on health systems and global cooperation. The wide variation in how PHC continues to be interpreted and operationalised risks undermining its transformative potential. Importantly, the consequences of these inconsistencies are felt at the country level. As noted by the European Commission, it is essential that the plurality of bodies active in global health are sufficiently aligned—avoiding duplication and fragmentation.^8^

To address this challenge, we aimed to review the current landscape and develop a clear understanding of how PHC is operationalised by major health and development organisations. To do so, we analysed the corporate strategy documents of a global sample of organisations to characterise the differences in the way that PHC is being operationalised with the goal of opening a cross-organisational dialogue and ultimately moving towards a more unified and coordinated approach to strengthen PHC globally.

## Methods

### Framework

We used the tripartite definition of PHC outlined in the 2018 Declaration of Astana^3^ and the related operational framework^9^ as the reference point for this study. According to this definition, PHC is a whole-of-society approach to health grounded in multisectoral policy and action and the empowerment of people and communities, with primary care and essential public health functions as the core of integrated health services. The Astana definition was applied because it received universal endorsement from all 193 UN member states at the 2018 Global Conference of Primary Health Care and builds on the seminal 1978 Alma-Ata Declaration.^1^ This definition was used as a lens to assess which elements of PHC are being most strongly emphasised by different organisations in their corporate strategy documents. This study adheres to the Consolidated Criteria for Reporting Qualitative Research.^10^

### Sample

We selected a purposive sample of major international organisations whose work intersects with the health and PHC space. We used PHC-focused partnerships and development assistance for health spending as subjective indicators.^11^, ^12^, ^13^ Three inclusion criteria were applied, counting each organisation once, based on the first criterion it met. First, we included all member organisations of the Sustainable Development Goal 3 Global Action Plan for Healthy Lives and Wellbeing for All (SDG3 GAP) and its PHC Accelerator (n=13). Second, we included all member organisations of the Universal Health Coverage Partnership (n=9). Finally, we consulted the top 25 contributors to development assistance for health, based on the latest Institute for Health Metrics and Evaluation Global Health Financing rankings.^13^

Our authorship team, affiliated with the WHO Special Programme for PHC, reviewed major international development organisations to identify those whose mandates or operations most prominently indicate their focus on the PHC approach (n=8). This purposive sampling reflected a consensus among the team based on professional judgement and experience, while acknowledging that other organisations might also contribute to PHC-related efforts in diverse ways. In total, 30 agencies were included, which we classified into three groups comprising 15 national ministries and agencies, nine UN organisations, and six other types of organisations (table 1).

**Table 1:** Organisations included in the review

	Type of organisation	Classification	Inclusion criteria met	Document
Agence Française de Développement [French Development Agency]	National agency	National	3	Strategic plan (2018–22)^14^∗
Belgian Ministry of Foreign Affairs, Foreign Trade and Development Cooperation	National ministry	National	2	Strategic plan (2021–24)^15^
Deutsche Gesellschaft für Internationale Zusammenarbeit [German Corporation for International Cooperation]	National agency	National	3	Strategic plan (2020–22)^16^
European Commission	Executive arm of the European Union	Other	2	Other strategy or plan^5^
French Ministry of Europe and Foreign Affairs	National ministry	National	2	Other strategy or plan (2023–27)^17^∗
Gates Foundation	Philanthropic foundation	Other	3	Other strategy or plan^18^
Gavi, the Vaccine Alliance	Public–private partnership	Other	1	Strategic plan (2021–25)^19^
German Federal Ministry of Health	National ministry	National	2	Other strategy or plan^20^
Global Affairs Canada	National agency	National	2	Other strategy or plan (2023–24)^21^
Global Financing Facility	Funding partnership	Other	1	Strategic plan (2021–25)^22^
Global Fund to Fight AIDS, Tuberculosis and Malaria	Financing and partnership organisation	Other	1	Strategic plan (2023–28)^23^
International Labour Organisation	UN organisation	UN	1	Strategic plan (2022–25)^24^
Irish Aid	National agency	National	2	Other report^25^
Japan International Cooperation Agency	National agency	National	3	Strategic plan (2017–21)^26^
Japanese Ministry of Health	National ministry	National	2	Other strategy or plan (2022)^27^
Joint UN Programme on HIV/AIDS	UN organisation	UN	1	No equivalent identified
Korea International Cooperation Agency	National agency	National	3	Strategic plan (2021–25)^28^
Luxembourg Development Agency	National agency	National	2	Other report^29^
Luxembourg Ministry of Foreign and European Affairs, Defence, Development Cooperation and Foreign Trade	National ministry	National	3	Strategic plan 2030^30^
Norwegian Agency for Development Cooperation	National agency	National	3	Strategic plan 2030^31^
UK AID	National agency	National	2	Strategic plan (2022)^32^
UN Children’s Fund	UN organisation	UN	1	Strategic plan (2022–25)^33^
UN Development Programme	UN organisation	UN	1	Strategic plan (2022–25)^34^
UN Population Fund	UN organisation	UN	1	Strategic plan (2022–25)^35^
UN Women	UN organisation	UN	1	Strategic plan (2022–25)^36^
Unitaid	Public–private partnership	Other	1	Strategic plan (2023–27)^37^
United States Agency for International Development	National agency	National	3	Strategic plan (2022–26)^38^
World Bank	UN organisation	UN	1	Other report (2023)^39^
World Food Programme	UN organisation	UN	1	Strategic plan (2022–25)^40^
WHO	UN organisation	UN	1	Strategic plan (2019–23)^41^

∗ These documents were identical in content and were treated as a single entity for the purpose of analysis.

### Search strategy and selection criteria

Most international development organisations generate large numbers of policy documents and technical reports annually. We aimed to analyse how PHC is operationalised in core corporate strategic documents, which are used to shape the work and vision of the organisation at its highest level. We explored the ways in which PHC is presented in the public-facing corporate strategy documents of each organisation.

In December, 2023, we accessed the official websites of each included organisation (n=30). The corporate strategy or plan was searched in a tailored approach that included reviewing the About the organisation page or equivalent or using the website’s dedicated search function with the terms “organization strategy” or “corporate strategic plan” or similar. When multiple strategic plans were available, the currently active plan—typically covering a 5-year timeframe—was prioritised, followed by, as relevant, the most recent preceding plan.

When an organisation-wide strategic plan was not available, the next most suitable document was downloaded using the following hierarchy: current strategic plan, then annual report, then global health strategy, then vision document, then PHC strategy. All reports were downloaded as PDF files and stored as a shared dataset available to all authors. The relevant files were cross-checked by the full study team for accuracy. For documents that were not available in English, we used Google Translate for translation and had a bilingual researcher review the English language version and correct any errors. We emailed an existing list of PHC or WHO focal points from all of the included organisations to confirm document selection. All documents were imported into NVivo 14 (Lumivero) for analysis.

### Analysis

We performed an abductive thematic content analysis^42^^,^^43^ to analyse how PHC was conceptualised in each of the documents. Content analysis is an approach that is used to identify the presence of specific words and themes within qualitative data. The conceptual content analysis approach was well suited for our study as it allows for a nuanced understanding of how PHC is being conceptualised and implemented across different organisational contexts. We performed both qualitative and quantitative analyses.

For qualitative analysis, each document was read and re-read so that we could familiarise ourselves with the data. Next, we used open coding for every mention of “primary health care”, “primary healthcare”, or “PHC”, followed by an iterative process of combining these open codes into themes, revisiting the data, and reviewing the themes. After this inductive coding phase, the themes were deductively grouped with reference to the three components of the PHC approach, per the definition of the Declaration of Astana presented in the Operational Framework for PHC.^9^ Finally, conceptual diagrams were used to map the different ways that PHC was being used in each of the strategy documents.

For quantitative analysis, every mention of “primary health care”, “primary healthcare”, and “PHC” in each document was counted, excluding those in the title, glossaries, table of contents, index, and in which the acronym PHC was introduced for the first time, as in “primary health care (PHC)”. Mentions of “primary care” were coded separately, as primary care is often treated as a concept distinct from PHC—eg, in the vision for PHC in the 21st century document that accompanied the 2018 Astana Declaration. Although these terms are widely used as synonyms, some agencies and health system researchers differentiate between them. Primary care is considered a service delivery platform (commonly characterised by first contact, comprehensiveness, coordination, continuity, and community centredness), whereas PHC is considered as a whole-of-society approach to health that includes all elements of the health system, plus empowered people and communities, and multisectoral policy and action. PHC-oriented health systems are characterised by a focus on equity, community engagement, prevention, multisectoral action, and strong primary care that are well integrated with other levels of the health system.^44^ To provide additional context, we also counted the number of mentions of “health” in each document.

### Member checking

On Nov 26, 2024, a 90-min virtual workshop was organised with PHC focal points from each of the organisations whose documents were included in the review. Participants were invited to the workshop based on an existing list of PHC focal points or health system leads or similar, with each organisation included in the sample. Some of these representatives appointed members of their team working most closely with the topic of the workshop. In total, 28 participants, representing ten different organisations, were present. The workshop included a presentation on the context of this study, the preliminary findings, and a moderated discussion led by members of the authorship team. Any corrections to the reports consulted in the review were made in January, 2025. The discussion during this session was recorded, and the transcript informed further reflections on the findings. This exercise confirmed the timeliness of the review, with many participants highlighting 2025 as a pivotal mid-decade moment for revising their corporate strategies and plans. Participants also affirmed the usefulness of the analysis, which helped to inform its refinement and anonymisation for broader dissemination.

### Reflexivity

This Health Policy paper was conceived following the authors’ (LNA, FK, and EB) previous observations of differing definitions of PHC and their observation that major development organisations were often conflating PHC with primary care service delivery, and emphasising different aspects of PHC when engaging with national policy makers.^7^ LNA is a British mid-career health systems researcher and family physician based at the University of Oxford (Oxford, UK). He consults for WHO and the World Bank and has previously worked with many of the organisations included in our sample. The other authors (EB, SS, SD, TT, and FK) are affiliated with WHO’s Special Programme on PHC, in which their work focuses on PHC-oriented policies, partnerships, research, and evidence synthesis. We have a diverse mix of gender, ethnicity, seniority, and clinical and non-clinical backgrounds in the authorship team.

### Anonymisation

Although WHO initiated and funded this study, the intention of the exercise was to generate mutual learning to open dialogue around constructive collaboration. We did not aim to police PHC terminology, create a new definition, or call out organisations for incorrect use or misuse of the term. Given that the core value of our findings lies in assessing the overall degree of alignment across organisations, rather than in picking out specific entities, we generated two versions of the findings: (1) an anonymised version for public dissemination and publication in peer-reviewed literature and (2) a de-anonymised confidential version that has been shared with all participating organisations to kickstart efforts to move towards greater consistency. This Health Policy paper presents the public-facing findings using three broad organisational categorisations: UN organisations, national agencies or ministries, and other organisation types. Direct quotes from the reviewed reports are cited by organisation type and document type (eg, organisation type: national; document type: strategic plan).

## Findings

### Strategic plans

Strategic plans were available for 22 (73%) of the 30 organisations. The global health strategy document was used for four organisations that did not have strategic plans, and annual reports or PHC-specific strategy documents were used for another three organisations. For one organisation, no appropriate document could be identified. We also found that the same strategic plan was used by two national organisations from the same country; so, these were treated as a single entity for the purpose of the analysis. As such, our analysis included 28 documents (all referenced in table 1).

### Frequency of PHC mentions

On average, the documents were 37 pages long (range: 2–164). 16 (57%) of the 28 documents had no mention of PHC at all, including all but two of the national agency or ministry documents (table 2). Document length is a possible reason for these omissions; for instance, two national development agency documents were only two and four pages long, focusing more on providing a broad overview of strategic priorities rather than granular detail on the strategic approach. Four of the included UN agency documents did not mention PHC in their strategy documents, which ranged in length from 9–49 pages and, on average, mentioned health on every other page.

**Table 2:** Frequency of mentions of PHC in each document reviewed by organisation type

	Organisation type	Number of mentions of PHC per page (mean)	Total number of mentions of PHC	Document length (number of pages)	Document type
Document 1	Other	10·3	6	62	PHC strategy or plan
Document 2	Other	0·47	8	17	Strategic plan
Document 3	UN	0·33	6	24	Strategic plan
Document 4	Other	0·25	8	32	Strategic plan
Document 5	Other	0·16	5∗	32	Other strategy or plan
Document 6	UN	0·12	3∗	25	Strategic plan
Document 7	UN	0·1	6†	60	Strategic plan
Document 8	National	0·07	2	38	Other strategy or plan
Document 9	National	0·04	3	68	Strategic plan and other strategy or plan
Document 10	Other	0·04	3	73	Strategic plan
Document 11	Other	0·02	1	52	Strategic plan
Document 12	UN	0·01	1	114	Other report
Document 13	National	0	0	81	Other strategy or plan
Document 14	National	0	0	52	Strategic plan
Document 15	UN	0	0	49	Strategic plan
Document 16	National	0	0	46	Other report
Document 17	National	0	0	44	Other strategy or plan
Document 18	National	0	0	34	Strategic plan
Document 19	National	0	0	28	Strategic plan
Document 20	UN	0	0	27	Strategic plan
Document 21	National	0	0	20	Strategic plan
Document 22	National	0	0	19	Other report
Document 23	National	0	0	13	Strategic plan
Document 24	UN	0	0	12	Strategic plan
Document 25	UN	0	0	9	Strategic plan
Document 26	National	0	0	4	Strategic plan
Document 27	National	0	0	3	Strategic plan
Document 28	National	0	0	2	Strategic plan

PHC=primary health care.

∗ Includes four mentions of “primary healthcare”.

† Includes one mention of “primary healthcare”.

12 (43%) of the 28 organisation documents mentioned PHC at least once (table 2). The frequency of mentions ranged from once every other page to once every 100 pages. One document mentioned PHC ten times per page; however, the document was a PHC strategy document (as we could not identify an overall corporate strategy document for the organisation under consideration). Formal definitions of PHC or primary care were not explicitly provided across all documents. Similarly, specific references or citations for these terms were generally not included.

### Conceptual use of PHC

**Figure 1:**
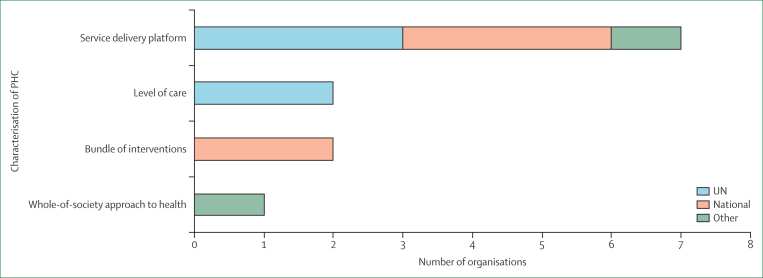
Representation of the four common conceptualisations of PHC PHC=primary health care.

Among the 12 documents that mentioned PHC at least once, we identified four different conceptualisations: PHC as a service delivery platform, a level of care, a bundle of interventions, and a whole-of-society approach to health (figure 1).

Only one document, from a national agency, conceptualised PHC as a whole-of-society approach to health grounded in the principles of equity, person-centredness, community, and integrated health systems. However, the same document went on to use PHC interchangeably with multiple other terms including “primary health services”, “primary health systems”, “primary and community health services”, “primary care”, and a “primary health care approach”. Most of these terms appear to be advancing the concept of primary care as a service delivery platform. The following is an example.

“COVID-19… disrupted access to primary health care.”

(Organisation type: national; document type: strategic plan)

This lack of internal consistency was common across most reviewed documents, with all but two using multiple conceptualisations of PHC. A consistent pattern was the use of PHC to refer to primary care, either as a setting or service delivery platform. Two organisations used PHC to characterise a specific bundle of services and the service delivery platform responsible for delivering these interventions.

“The [organisation] is designed to help governments ramp up provision of a broad scope of quality, affordable primary health care services critical for improving the health and nutrition of women, children and adolescents—including, but not limited to, family planning services, antenatal care, obstetric care, services to prevent stillbirth, ….”

(Organisation type: other; document type: strategic plan)

“...extending immunisation services to reach [unimmunised children] can also serve as a platform for countries’ integrated delivery of other PHC services, laying a solid foundation for progressively universalising PHC in support of universal health coverage.”

(Organisation type: other; document type: strategic plan)

Although two documents referred to primary care service delivery “systems” and others used the term “platforms”, both usages operationalised PHC in the same way. Hence, we coded them all as service delivery platforms—a set of components that provide a service delivery architecture (ie, primary care services). We distinguished between PHC as a platform and as a level of care when the level-of-care term was used to distinguish primary care from hospital-based services.

“The immediate push will be to accelerate access to optimal [treatment] packages for main causes of death, such as TB, cryptococcal meningitis, and severe bacterial infections, at the primary health care level.”

(Organisation type: other; strategic plan)

Several agencies used additional descriptors (eg, community governance, a focus on equity and marginalised groups, and person-centredness) to characterise primary care services in ways that intersected with elements of community engagement or empowerment. One document from the Other category nested PHC within core public health functions. However, the core aspect of the PHC definitions was firmly rooted in the third element of the Astana tripartite PHC conceptualisation—integrated health services with an emphasis on primary care.

It was clear from the documents that PHC is broadly seen as a means of delivering clinical services in the local community. Many documents used the term “integrated” to characterise PHC services, mainly pertaining to the delivery of multiple interventions (eg, antihypertensive medication plus infectious disease immunisations) rather than integration across providers or levels of care; however, one exception was found in a document of a UN organisation.

Although most organisations used PHC as a synonym for primary care services, a substantial variation was observed in how these services were perceived. As noted, primary care is typically characterised as follows: the first point of contact with the health system for all but emergency care, being located in or close to communities and engaging them as partners, offering a comprehensive range of services that meet all of the common needs of the local population, coordinating patients’ care across different providers and levels of the health system, and providing person-centred and continuous care over time. Those documents that provided more than a cursory mention of PHC tended to stress first contact, proximity to communities, and delivery of multiple interventions, generally badged as the delivery of integrated services. However, the strategic role of primary care services in delivering coordinated, continuous, and comprehensive care was rarely emphasised.

Three different interpretations of PHC were obtained from the analysis. The first was PHC as a whole-of-society approach to health*,* comprising all components of the health system, including primary care, multisectoral action, and community engagement and empowerment. This version is articulated in one of the national documents and resonates with much of the language and principles in the strategy documents from UN and other partner organisations. The second interpretation characterises PHC as strong or high-quality primary care and centres on the primary care service delivery platform emphasising first contact, comprehensiveness, continuity, coordination, and community-centred care, often delivered by multidisciplinary teams. The third version that emerged from the documents could be characterised as first-contact community-based services, or basic primary care, providing a targeted set of community-based interventions, without necessarily ensuring care coordination or continuity. Although these conceptualisations differ, they are frequently referred to using the same term—PHC—leading to potential misalignment in interpretation and implementation across organisations.

## Discussion

30 global organisations with a clear development assistance for health agenda conforming with the principles of PHC were identified. Fewer than half of the strategic documents reviewed from these organisations mentioned PHC. Among those that did, a reasonable level of consistency was observed, with most documents operationalising the term PHC as a form of primary care—specifically, a first-contact, community-based platform to deliver a defined set of essential health services that meet the most pressing needs of the local community.

This alignment is encouraging, although there is some variation in the strength of the primary care platform being advanced by each organisation. This approach to capturing primary care is not inherently right or wrong. However, it is important that organisational leads reach consensus around which elements of the primary care delivery platform are most important and focus their efforts accordingly. For instance, should primary care be resourced to deliver basic, essential, or comprehensive services? Consistency is needed across senior leadership and those responsible for relevant departments and programmes within organisations and across organisations.

Several strategy documents mentioned themes that are central to PHC, including person-centredness, community centredness, equity, participatory governance, and the integration of different parts of the health system around the needs of people and places. However, with the exception of one national strategy, these themes are not being used with reference to PHC. Rather, the themes are discussed as standalone health system goals without an overarching conceptual narrative. The conflation of PHC with primary care service delivery poses the risk that funding and support earmarked for broad PHC efforts—including multisectoral action, community engagement, and wider health system integration—might be inappropriately channelled towards clinical health-care provision. Given the varied interpretations, funders and policy makers should take additional care to specify which components of PHC they are intending to target under the banner of PHC strengthening.

We identified scarce reference to primary care coordination, continuity, and multisectoral action. These are foundational concepts for primary care and the broader concept of PHC that do not seem to feature in the current global approach. Hence, discussion is warranted to explore the extent to which this absence is intentional or justified.

This situation is, however, not static. WHO, for example, has actively worked to address the challenge of putting PHC into practice. Comparing WHO’s Thirteenth (2019–23)^41^ and Fourteenth General Programme of Work (2025–28)^45^ revealed an evolution in the right direction. The new programme of work aligns more closely with the original vision of the Alma-Ata and Astana Declarations by positioning PHC as an approach, rather than equating it solely to a level of care. This shift, although encouraging, highlights the difficulty faced by global health organisations in maintaining fidelity to the intended meaning of PHC. The changing global health landscape introduces a further challenge to global alignment, as the very principle of collective action is being attacked.

**Figure 2:**
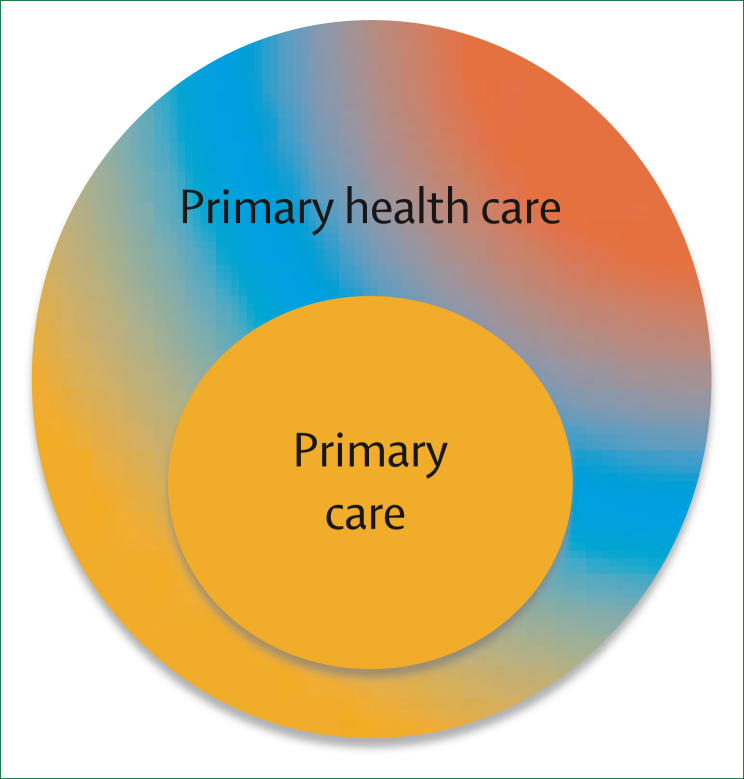
Representation of the distinction between primary health care and primary care, wherein primary care is one aspect of the larger primary health care approach

Our engagement with the international organisations included in our sample suggests a genuine enthusiasm to work towards a consistent PHC approach. To support this progress, we encourage development partners and the broader global health community to be guided by the WHO member state-endorsed vision of PHC as an approach (large circle in figure 2) and avoid oversimplifying its meaning to primary care as a service delivery platform (smaller circle in figure 2). Although primary care often serves as the entry point for many health system reforms, an overly narrow focus on primary care risks underinvestment in the other two key components of PHC—namely, multisectoral policy and action and empowered people and communities.

In 2025, many organisations are preparing to update their corporate and national strategies. This mid-decade moment, coupled with the countdown to 2030 global health targets and the implementation of the Lusaka Agenda, offers an opportunity for recalibration of our approach. The shifting geopolitical landscape that is reshaping official development assistance demands further alignment on global health priorities and cooperation. An interagency statement on PHC could serve as a tool to promote a more cohesive and multilateral stance on the elements of PHC that should be prioritised on the path to 2030. Such a statement could potentially encourage greater alignment of partner support to countries as they work to reorient health systems towards the PHC approach.

### Limitations

In this Health Policy paper, we used a robust qualitative approach for data collection and analysis; however, independent double coding of documents could not be performed owing to time and resource constraints. The list of partner organisations, despite including all the major actors in the PHC space, cannot be considered as definitive because the criteria used to circumscribe this group were subjective. Furthermore, the documents included in the analysis were not all equivalent, and substantial heterogeneity in style and length was observed even among corporate strategy documents. Therefore, the findings of this analysis should be considered a first step in exploring how international organisations are operationalising PHC.

Although we found that only one organisation is (partially) operationalising PHC as a whole-of-society approach, future research could explore whether there are differences in real-world operations and impact according to how different agencies conceptualise this important concept.

## Conclusion

Highly influential partner organisations often use similar language and concepts to operationalise PHC in their strategic plans. However, our analysis revealed that current usage is poorly aligned with the expansive definitions of PHC found in the Astana and Alma-Ata declarations. Across these declarations, PHC is considered to be an approach to health that includes all aspects of the health system (and beyond), whereas in the documents reviewed, PHC is almost always used as a direct synonym for primary care. To stay committed to the holistic principles of PHC, we must maximise its potential to improve global health outcomes and achieve universal health coverage, and the opportunity for realignment in 2025 should not be missed. Moving forward, collaborative dialogues could enhance a further shared understanding of how to operationalise PHC in practice as the whole-of-society approach intended.

## Data sharing

The full dataset is available upon reasonable request to the corresponding author.

## Declaration of interests

FK, SS, TT, and SD are staff members of WHO. The authors alone are responsible for the views expressed in this publication. The views in this manuscript do not necessarily represent the views, decisions, or policies of WHO. LNA and EB declare consultancy fees from WHO. LNA also declares consultancy fees from the World Bank, although these fees were not associated with the current work.
